# Bone Marrow Stem Cell Population in Single- and Multiple-Level Aspiration

**DOI:** 10.3390/biomedicines12122731

**Published:** 2024-11-28

**Authors:** Xiangguo Che, Hee-June Kim, Xian Jin, Joon-Woo Kim, Kyeong-Hyeon Park, Jeong-Ok Lim, Hee-Soo Kyung, Chang-Wug Oh, Je-Yong Choi

**Affiliations:** 1 Department of Biochemistry and Cell Biology, Cell and Matrix Research Institute, School of Medicine, Kyungpook National University, Daegu 41944, Republic of Korea; xiangguo0622@naver.com (X.C.); kimhan911021@naver.com (X.J.); 2 Department of Orthopedic Surgery, Kyungpook National University Hospital, School of Medicine, Kyungpook National University, Daegu 41944, Republic of Korea; june0104@daum.net (H.-J.K.); orthopedics@naver.com (J.-W.K.); jolim@knu.ac.kr (J.-O.L.); hskyung@knu.ac.kr (H.-S.K.); 3 Severance Children’s Hospital, Yonsei University College of Medicine, Seoul 03722, Republic of Korea; pkh1112@gmail.com; 4 Joint Institute for Regenerative Medicine, Kyungpook National University, Daegu 41940, Republic of Korea; 5 Bio-Medical Research Institute, Kyungpook National University Hospital, Daegu 41940, Republic of Korea

**Keywords:** bone marrow aspiration concentrate, mesenchymal stem cells, single level, multiple levels, musculoskeletal injuries

## Abstract

Background: Bone marrow aspiration concentrate (BMAC) has garnered increasing interest due to its potential for healing musculoskeletal injuries. While the iliac crest remains a common harvest site, the aspiration technique’s efficacy in offering the highest yield and prevalence of mesenchymal stem cells (MSCs) is controversial. This study aimed to compare two different techniques of bone marrow aspiration over the anterior iliac crest from a single level versus multiple levels. Methods: Anterior iliac crests were selected in seven adult patients (aged between 31 and 59 years old). Aspiration was achieved using an 11-gauge needle (length: 100 mm; diameter: 2.3 mm) specifically manufactured for bone marrow collection (BD, Becton, Franklin Lakes, NJ, USA) connected to a 10 mL syringe. On one side, 4cc of bone marrow was aspirated at a single level to a depth of 7 cm without changing the needle direction. On the other side, over the same portion of the iliac crest, 1 cc of bone marrow was obtained from multiple levels of different depths during needle retrieval, maintaining a distance of 1 cm and changing the tip direction. The samples were blindly sent to the laboratory without indicating whether they came from an single level or multiple levels. Fluorescence-activated cell sorting (FACS) and osteoblast differentiation were analyzed and compared. Results: In the FACS analysis, the single level resulted in a higher population of MSCs that were positive for CD105, CD73, and CD90 and negative for CD34, compared to the multiple-level method. In the process of osteoblast differentiation, it was observed that MSCs exhibited more advanced features of enhanced osteoblastic abilities in the single-level method rather than the multiple-level method. Conclusions: A single-level aspiration technique at the anterior iliac crest may produce a high-quality bone marrow aspirate. This technique may help obtain specific populations of MSCs with the desired characteristics for use in regenerative therapies for musculoskeletal injuries.

## 1. Introduction

The musculoskeletal system plays a vital role in the human body, serving essential functions that involve both structural and metabolic functions. It supports structure, facilitates movement, protects internal organs, and contributes to metabolic processes related to mineral storage and blood cell production [1,2]. Musculoskeletal injury, the most common cause of disability and daily life limitations, presents a significant socioeconomic challenge [3,4,5].

Regeneration of musculoskeletal injuries poses challenges in patients with extensive tissue damage, disease, and aging with reduced tissue regeneration capacity. Mesenchymal stem cell (MSC)-based therapy could be an option for repairing and regenerating musculoskeletal tissues. For example, osteoblasts produce a substantial volume of macromolecules in the bone matrix, including collagenous and noncollagenous proteins that provide a scaffold for matrix mineralization through the deposition of calcium phosphate in the form of hydroxyapatite. Moreover, osteoblasts specialize in matrix production and mineralization for the formation of strong bones. In a mouse model, the origins of osteoblasts include chondrocytes in the growth plate, quiescent bone-lining cells on the bone surface, specialized fibroblasts in the craniofacial structures, and MSCs in the bone marrow. Particularly, MSCs in the bone marrow are associated with bone development, regeneration, bone anabolism, and pathological ossification [6].

Bone marrow aspiration concentrate (BMAC) has become one of orthopedics’s most common cell-based musculoskeletal injury therapies [7,8]. BMAC has the characteristic of being a rich source of pluripotent MSCs and growth factors. Moreover, BMAC has anti-inflammatory and regenerative effects for treating musculoskeletal diseases [9,10]. Many reports have highlighted using BMAC in various orthopedic fields, including osteonecrosis [11], nonunion of bone fracture [12], bone defects [13], and cartilage repair [14].

In biological MSC-based therapy, the primary goal is to improve the methods for obtaining high concentrations of MSCs [15]. Autologous bone marrow obtained from iliac crest bone marrow aspiration has been one of the most utilized sources for cell-based therapies in orthopedics [16]. BMAC contains MSCs, red blood cells, white blood cells, platelets, hematopoietic cells, and nonhematopoietic precursors. The MSC population in BMAC is very small, typically estimated to be 0.01–0.02% of the total cell volume [15,17]. However, the aspiration technique for offering the highest yield is controversial, and the prevalence of MSCs remains obscure. There are various methods for obtaining bone marrow, but a consensus still needs to be reached. Because the aspiration volume can affect the number of MSCs, the method of aspiration at a single or multiple levels may affect the concentration and quality of the MSCs.

In this study, it was hypothesized that if bone marrow were aspirated from at multiple levels, more MSCs could be obtained due to each new aspirated bone marrow compared to bone marrow aspirated in a single level. The purpose of this study was to compare the concentration of MSCs and their potency of BMAC in two different techniques of bone marrow aspiration over the anterior iliac crest from the single level versus the multiple level using fluorescence-activated cell sorting (FACS) analysis and osteogenic differentiation.

## 2. Materials and Methods

### 2.1. Antibodies and Reagents

PE-CyTM7 Mouse Anti-Human CD73, APC Anti-Human CD90, FITC Mouse Anti-Human CD105, and PE Mouse Anti-Human CD34 were purchased from BD Biosciences. Fetal bovine serum and Type IV collagenase were purchased from Gibco (Grand Island, NY, USA), ascorbic acid, β-glycerophosphate, and Alizarin red were purchased from Sigma-Aldrich (St. Louis, MO, USA), and Dulbecco’s modified Eagle’s medium (DMEM) was purchased from Lonza (Rockland, ME, USA).

### 2.2. Human Subjects

This study enrolled 7 Korean patients aged 30 to 60 years. The participants were undergoing orthopedic surgery and had no history of diabetes, blood disorders, hematological malignancies, or use of immunosuppressive medications or medications with bone marrow–suppressive effects. The Institutional Review Board of Kyungpook National University Hospital approved using human bone marrow biopsies (IRB File No of KNUH 2021-12-028). Written informed consent documentation was obtained from all patients before the surgical procedure. The human sample list is described in Table 1.

### 2.3. Bone Marrow Aspiration

**Figure 1:**
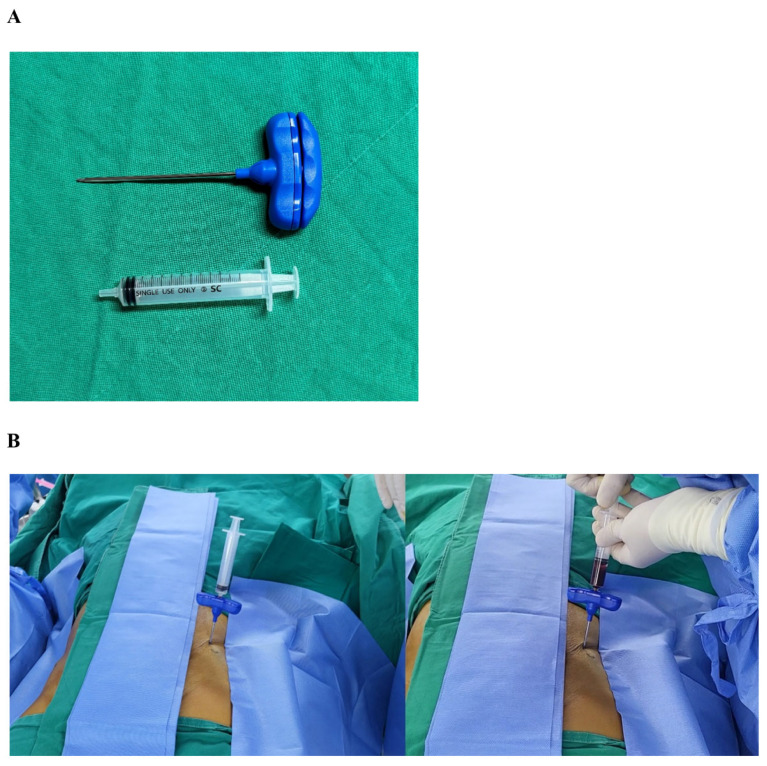
The bone marrow aspiration tool. (**A**) The bone marrow aspiration tool with an 11-gauge needle specifically manufactured for bone marrow collection and a 10 mL syringe. (**B**) Aspiration was achieved using the aspiration tool. The needle was inserted into the anterior iliac crest and connected to a 10 mL syringe.

Bone marrow cells were isolated from the anterior iliac crest using single- or multiple-level needle puncture aspiration from 9 male patients (mean age 46.7 ±11.8 years, range 30 to 60 years) after receiving written informed consent. Briefly, bone marrow aspirates (4 mL) were collected with the syringe with anticoagulant (10 U/mL heparin), and the Ficoll-Paque (1.078 g/mL) method was used for cell separation. Anterior iliac crests were selected in 7 adult patients for marrow aspiration sites. Aspiration was achieved with an 11-gauge needle (length, 100 mm; diameter, 2.3 mm) specifically manufactured for bone marrow collection (BD, Becton, Franklin Lakes, NJ, USA) connected to a 10 mL syringe (Figure 1).

**Figure 2:**
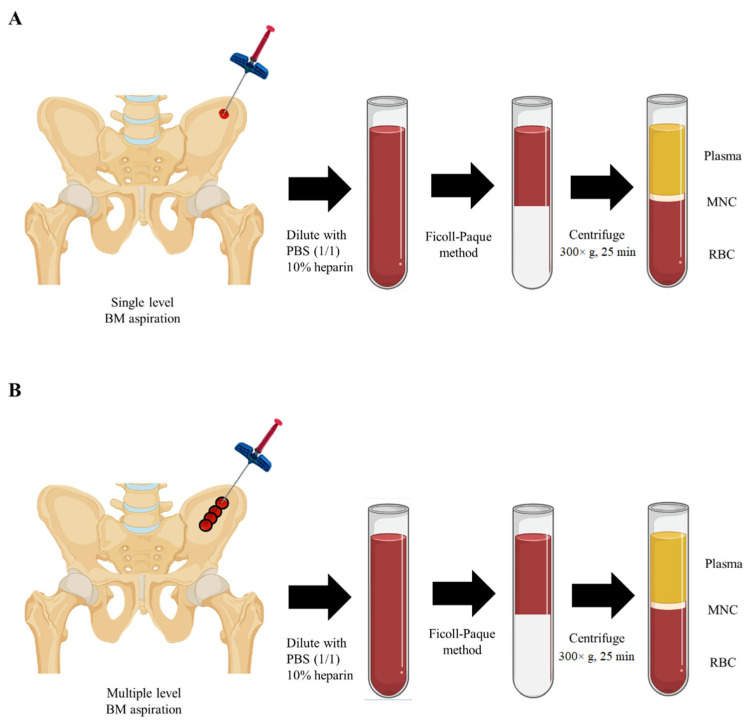
Illustrations of single-level and multiple-level methods for bone marrow aspiration. Bone marrow cells were isolated from the iliac crest through needle puncture aspiration, utilizing a single level or multiple levels, and obtained bone marrow cells were separated using the Ficoll-Paque method. (**A**) In single-level samples, 4 mL of bone marrow was aspirated at a depth of 7 cm using a single needle insertion without altering its direction. (**B**) In multiple-level samples, 4 mL of bone marrow was aspirated from multiple depths withdrawn at intervals of 1 cm, changing the tip direction by 180 degrees with each withdrawal obtained for each 1 mL of bone marrow on the other side of the iliac crest. Abbreviations: BM: bone marrow; PBS: phosphate-buffered saline; MNC: mononuclear cell; RBC: red blood cell.

In single-level samples, 4 mL of bone marrow was aspirated at a single level of a 7 cm depth without changing the needle direction. In multiple-level samples, on the other side over the same portion of the iliac crest, each 1 mL of bone marrow was gained at multiple depths while retrieving the needle at a distance of 1 cm and changing the tip direction by 180 degrees. A total of 4 mL of aspirate was obtained (Figure 2). The samples were blindly sent to the laboratory without the notice of the methods.

### 2.4. Fluorescence-Activated Cell Sorting

Bone marrow cells obtained through single- or multiple-level isolation methods were characterized by their mesenchymal cell phenotype. Before fluorescence-activated cell sorting (FACS) analysis, MNCs were separated using a Ficoll-Paque (1.078 g/mL) separation solution through centrifugation at 400× *g* for 25 min at 4 °C, without applying a brake. The separated MNCs were then washed three times with a FACS buffer containing 2.5% BSA and 0.25% sodium azide (Sigma) in PBS. The cells were incubated for 1 h at 4 °C with the following markers: positive markers CD73 (PE-CyTM7-conjugated), CD90 (APC-conjugated), and CD105 (FITC-conjugated), as well as the negative marker CD34 (PE-conjugated). After incubation, the cells were briefly washed in a FACS buffer. FACS data were acquired using FACS Calibur II (BD Biosciences). For FACS analysis, MNCs obtained from bone marrow aspiration via single- or multiple-level methods were gated to 1 × 10⁶ cells, and surface markers were detected using 488 nm or 640 nm lasers.

### 2.5. Cell Seeding and Osteogenic Differentiation

For osteogenic differentiation, bone marrow-derived mononuclear cells (MNCs) were directly seeded in the 24-well plate at a density of 1 × 10^4^/cm^2^ after separation from the bone marrow and incubated in an osteogenic medium containing an alpha-minimum essential medium (α-MEM, Gicbo BRL, Gaithersburg, MD, USA) supplemented with 10% fetal bovine serum (FBS, Gibco BRL, Gaithersburg, MD, USA), 100 nM of dexamethasone (Sigma-Aldrich, St. Louis, MO, USA), ascorbic acid (50 μg/mL), and beta-glycerophosphate (10 mM) for osteoblast differentiation [18]. MSCs were cultured in an osteogenic medium for 28 days with a medium change 3 times a week.

### 2.6. Alizarin Red Staining

Osteoblast mineralization was assessed using Alizarin red staining. After 28 days of osteogenic differentiation, the differentiated cells were washed 3 times with PBS, fixed with 4% paraformaldehyde (PFA) for 10 min at room temperature, and washed 3 more times with PBS. The cells were then stained with 2% Alizarin red solution (pH 4.2) for 20 min, followed by 3 washes with distilled water. Calcium deposition was imaged using a Leica microscope and analyzed using the Bioquant Osteo 2019 v19.9.60 program (Bioquant Osteo, Nashville, TN, USA).

### 2.7. Statistical Analysis

Statistical analyses were performed by GraphPad Prism 9 (GraphPad, San Diego, CA, USA), and data were expressed as mean ± standard deviation. Statistical significance was analyzed using a Student’s t-test and indicated as follows: * *p* < 0.05, *** *p* < 0.01.

## 3. Results

### 3.1. Advanced MSC Population in a Single-Level Bone Marrow Aspiration

**Figure 3:**
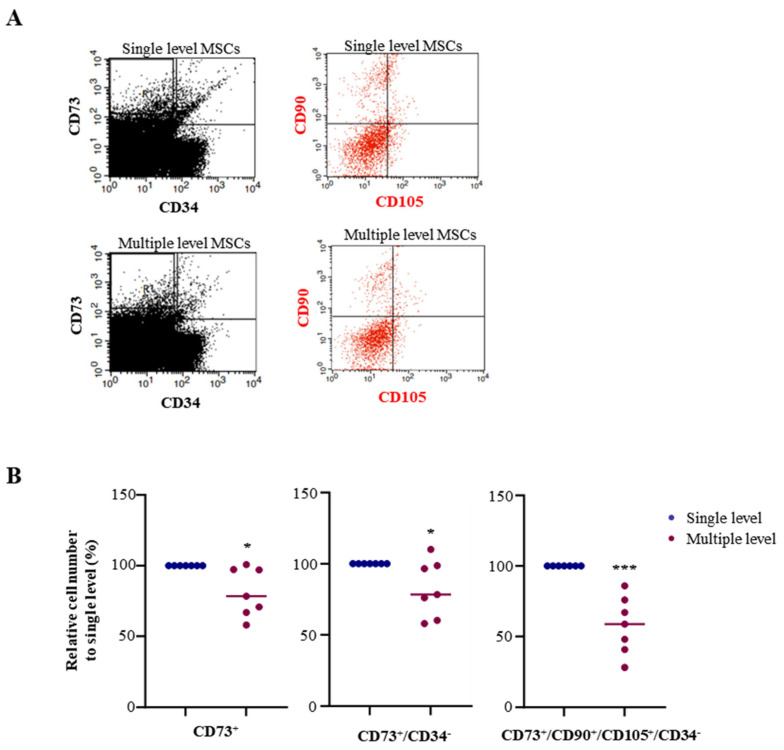
MSC population of samples from a single level and multiple levels. Fluorescence-activated cell sorting (FACS) analysis of the expression of surface antigens on MNCs isolated from single-level or multiple-level bone marrow aspiration. A minimum amount of 1 × 10^6^ cells was examined for the FACS analysis. (**A**) The CD73^+^ and CD34^−^ expression MNCs were measured and gated the cells for the specific co-expression of CD73^+^ and CD34^−^ cells (black panel). The gated cells were examined for co-expression for surface antigens CD90 and CD105 and were counted as a pure MSC population (red channel). (**B**) The CD73^+^, CD73^+^/CD34^−^, and CD73^+^/CD90^+^/CD105^+^/CD34^−^ cells were counted using FACS analysis. The percentage was calculated by single/single and multi/single ratios. Abbreviations: MSC: mesenchymal stem cell. * *p* < 0.05, *** *p* < 0.01.

Separate MSCs were analyzed using FACS assay to assess the MSC population in single and multiple levels using bone marrow aspiration methods. For quantification, the high purity of MSC population CD73 (PE-CyTM7-conjugated), CD90 (APC-conjugated), and CD105 (FITC-conjugated) was used as a positive marker, while CD34 (PE-conjugated) was used as a negative marker in FACS analysis (Figure 3A). First, the number of CD73^+^ cells from single and multiple levels of bone marrow aspiration was analyzed using FACS. FACS analysis of the bone marrow cells obtained from each group revealed that multiple-level bone marrow aspiration significantly decreased CD73 to 81.3% compared to single-level bone marrow aspiration. To exclude hematopoietic stem cells and adipose tissue-derived MSCs, the cells were identified as CD73-positive and CD34−negative using FACS analysis. Compared with single-level bone marrow aspiration, multiple-level bone marrow aspiration significantly decreased CD73^+^ and CD34^−^ to 82.6%. Most reports identify CD73^+^/CD34^−^-expressing cells as MSCs in bone marrow, but MSCs are not the only cells expressing CD73^+^/CD34^−^. As B cells mature, CD73 expression increases, and CD34 expression decreases, yet mature B cells also express CD73^+^/CD34^−^ in bone marrow. Therefore, CD73^+^/CD34^−^-expressing cells do not accurately reflect the MSC count. To address this, MSCs were identified using CD90^+^/CD105^+^ markers to better quantify the pure MSC population. FACS analysis was employed to gate cells that were CD90+ and CD105+ within the CD73^+^/CD34^−^ population. The number of CD90^+^ and CD105^+^ among CD73^+^ and CD34^−^ cells in multiple-level bone marrow aspiration significantly decreased to 57.8% compared with single-level bone marrow aspiration (Figure 3B, Table 2).

### 3.2. Advanced Osteoblast Mineralization in Single-Level MSCs

**Figure 4:**
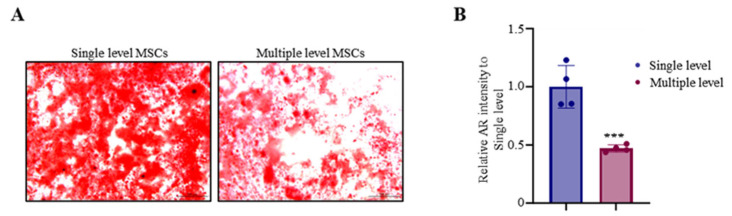
Advanced osteoblast mineralization in single-level MSCs. Harvested MNCs derived from a single level and multiple levels were used for osteoblast differentiation. Osteoblast mineralization was confirmed by the (**A**) Alizarin red staining and (**B**) quantified by the Bioquant Osteo 2019 v19.9.60 program at day 28 after osteogenic differentiation. Abbreviations: AR: Alizarin red; MSCs: mesenchymal stem cells; single level: a single level; multiple levels: multiple levels; *** *p* < 0.01.

Next, to assess the osteogenic differentiation ability of MNCs derived from single- and multiple-level aspiration, Alizarin red staining was performed. Alizarin red analysis of the bone marrow cells obtained from each group revealed that multiple-level bone marrow aspiration significantly decreased bone mineralization to 47% compared to single-level bone marrow aspiration. Mesenchymal stem cells of single-level bone marrow aspiration were more rapidly mineralized and differentiated into osteoblasts than those of multiple-level bone marrow aspiration in Alizarin red staining (Figure 4A). The quantification of osteoblast mineralization areas was also significantly enhanced in a single level compared with multiple levels (Figure 4B, Table 3). These results indicate that single-level bone marrow aspiration has promoted ability in osteogenic differentiation.

## 4. Discussion

This study found that bone marrow aspiration from a single level showed a higher population of MSCs than those of a multiple-level one. In osteoblast differentiation, MSCs exhibited more advanced features of osteoblastic abilities in the single-level rather than the multiple-level method. These results indicate that the single-level bone marrow aspiration showed a higher yield and prevalence of MSCs and more advanced osteoblastic differentiation than the multiple-level technique. The changes in depth during aspiration could affect the concentration and quality of MSCs and it is thought that the concentration of MSCs varies depending on the depth and is lower when performing aspiration BM at a shallower depth.

In orthopedic surgery, MSCs could be options for regenerative treatment in various problems, and several studies are ongoing. MSCs enable the regeneration of musculoskeletal tissues, especially bone marrow stromal cells (BMSCs), which can differentiate into several lineages, including chondrocytes, osteoblasts, and adipocytes [7]. BMSCs were commonly used sources of MSCs because of the easy collection method. Although BMSCs can be used as a cell suspension after being expanded by culture or as a concentrate, the clinical practice may be limited, considering the problems related to cell manipulation and expansion. However, BMAC can overcome the need for culture expansion, and minimal manipulation can be applied in a one-step treatment. It has been widely utilized in the clinical practice of orthopedic areas. It also could prevent the risk of allogenic disease and infection. Therefore, the clinical use of BMAC will expand further.

MSCs from BMAC could be applied in various areas of orthopedics. First, it could be used in the treatment of osteonecrosis. Hernigou et al. [17] suggested that ONFH may be the disease of bone cells and/or MSCs. The numbers and activity of MSCs decreased in bone marrow in patients with ONFH. Moreover, the number of progenitor cells in patients who underwent corticosteroid treatment was lower than in patients with a different underlying etiology. In this situation, BMAC could be a treatment option. Autologous MSCs exhibit anti-inflammatory, immunomodulatory, and tissue-protective “trophic” effects [19]. BMAC also applies to osteoarthritis (OA) patients, especially for that in the knee [20]. The treatment options were various in patients with knee OA. From non-pharmacological treatment, including weight loss and physical treatment, to pharmacological treatment, including oral medication and intra-articular injection, the various options can be applied by non-surgical treatment [21]. Recently, the treatment using MSCs has been considered the new treatment option for OA due to its structural contributions to tissue repair, its immunomodulatory and anti-inflammatory actions through direct cell-to-cell interaction, or the secretion of bioactive factors. Finally, BMAC could be used in the treatment of fractures and nonunion. In the musculoskeletal system, bone is a dynamic self-healing tissue that continuously remodels in response to mechanical loadings throughout life. However, bone remodeling cannot repair between 5 and 10% of all bone fractures, resulting in delayed or failed healing in extensive traumatic injuries [22,23]. In clinical practice, the preferred approach for treating fractures is autologous iliac crest bone graft replacement, which is considered the gold standard for treatment [24]. However, the application limitation was the significant bone defect, and the donor site morbidity could be a problem after an autologous bone graft. The other methods for nonunion, including allograft, bone substitutes, demineralized bone matrix (DBM), and ultrasound and pulsed electromagnetic field, could be used for bone healing. Osteogenic stem cells in bone marrow have the biological efficacy of cancellous bone [25,26]. Therefore, they would be applied to the treatment of fracture or nonunion [27,28].

In biological MSC-based therapy, the primary goal is to improve the methods for obtaining high concentrations of MSCs. Autologous bone marrow obtained from iliac crest bone marrow aspiration has emerged as one of the most utilized sources for cell-based therapies in orthopedics. BMAC contains MSCs, red blood cells, white blood cells, platelets, hematopoietic cells, and nonhematopoietic precursors. The MSC population in BMAC is rare and is typically estimated to be 0.01–0.02% of the total cell volume [15,16]. The osteogenic capacity correlated with the cell concentration [29] and the efficacy could be related to the number of progenitors [16]. Therefore, efforts to increase the progenitor cells and maximize the MSC content at the time of the aspiration are essential, and accurate methods for bone marrow aspiration are very important for promising therapeutic options for orthopedic treatments. There still needs to be a consensus on the BMAC aspiration method. Numerous studies have concentrated on enhancing bone regeneration by applying MSC-based therapy. Oliver et al. [30] reported no significant difference in cell concentration between single- and multi-site bone marrow aspiration systems, potentially due to limited sample analysis. However, their results showed a trend toward a 30% increase in BMSC yield with the single-site method compared to the multi-site method. They also mentioned that the single-site technique was associated with reduced pain, making it more acceptable to patients. In this study, the single-level bone marrow aspiration technique obtained a higher population of MSCs with MSC positive markers of CD73, CD90, and CD105, while there were negative MSC markers of CD34 compared with those of the multiple-level bone marrow aspiration technique. It is thought that changes in position, depth, and direction during aspiration affect the concentration and quality of MSCs. Obtaining a high-quality bone marrow aspirate for clinical application could be helpful.

The standardized measurement and reporting on the yield and quality of bone marrow aspirate samples, the colony-forming unit, and the combination of different markers have been suggested to characterize MSCs present within BMAC [31]. The International Society for Cellular Therapy (ISCT) has established standardized terminology and minimal mesenchymal stem cell (MSC) characterization criteria. According to these criteria, MSCs must possess plastic-adherent properties in standard in vitro culture systems, express surface markers CD73, CD90, and CD105, and lack the expression of hematopoietic markers CD34, CD45, CD14, CD19, and HLA-DR. Additionally, MSCs should demonstrate the ability to differentiate into osteoblasts, adipocytes, and chondroblasts in vitro [31,32]. Our data determined that the single-level bone marrow aspiration technique obtained a higher population of MSCs with MSC-positive markers of CD73, CD90, and CD105 and negative MSC markers of CD34 compared with the multiple-level bone marrow aspiration technique.

The limitation of this study was its small number of patients. The volume of BM aspirate, the size of the syringe, and the pressure during aspiration could change the results. However, this study evaluated only the aspiration method between single and multiple levels. Further larger-scale studies considering various methods of aspiration should be evaluated.

Obtaining a higher yield of bone marrow stem cells from sheep bone marrow is critical for musculoskeletal regeneration. However, the optimal method for achieving this remains unclear, with strong arguments on both sides. Our findings highlighted that single-level bone marrow aspiration is more efficient than the multi-level method for obtaining bone marrow stem cells while reducing patient damage and pain during the collection process. These results could be valuable for clinical autologous cell collection using bone marrow stem cells.

## 5. Conclusions

A high-quality bone marrow aspirate could be obtained through a single-level aspiration technique at the anterior iliac crest rather than a multiple-level aspiration technique. This approach is known for its effectiveness in obtaining specific populations of mesenchymal stem cells with the desired characteristics, making them valuable for regenerative therapies for musculoskeletal injuries.

## Figures and Tables

**Table 1:** The human sample list.

Patients	Age	Race	Location of Aspiration
1	31	Asian	Iliac crest
2	31	Asian	Iliac crest
3	58	Asian	Iliac crest
4	58	Asian	Iliac crest
5	41	Asian	Iliac crest
6	58	Asian	Iliac crest
7	45	Asian	Iliac crest

**Table 2:** Relative MSC numbers of multiple levels compared with a single level.

Markers	Relative MSC Number to Single Site (%)	*p*-Value
CD73^+^	81.3 ± 17.04	<0.05
CD73^+^/CD34^−^	82.6 ± 19.92	<0.05
CD73^+^/CD90^+^/CD105^+^/CD34^−^	57.8 ± 20.29	<0.001

**Table 3:** Relative Alizarin red intensity of samples from multiple levels compared with a single level.

	Relative AR Intensity to a Single Site (%)	*p*-Value
Compared with multiple sites	47 ± 17.04	<0.05

AR: Alizarin red.

## Data Availability

Data are provided within the manuscript.
